# Schisandrin B Exerts Radiosensitizing Effects on Breast Cancer via Dual Mechanisms of Cell Cycle/DNA Repair and Gut Microbiota-Immune Axis Modulation

**DOI:** 10.3390/ph19060883

**Published:** 2026-06-01

**Authors:** Yanhua Fang, Mengxuan Wang, Man Tong, Yue Wang, Zeshuo Feng, Ruoyu Wang, Zhe Wang, Lingyun Jia, Shanshan Liang

**Affiliations:** 1 Liaoning Key Laboratory of Molecular Recognition and Imaging, School of Bioengineering, Dalian University of Technology, Dalian 116024, China; fangyanhua@s.dlu.edu.cn; 2 The Key Laboratory of Biomarker High Throughput Screening and Target Translation of Breast and Gastrointestinal Tumor, Affiliated Zhongshan Hospital of Dalian University, No. 6 Jiefang Street, Zhongshan District, Dalian 116001, China; mengxuan_wang@126.com (M.W.); tongman928@163.com (M.T.); wydsghtc@163.com (Y.W.); fengzeshuo0610@163.com (Z.F.); wangruoyu@dlu.edu.cn (R.W.); wangzhe@dlu.edu.cn (Z.W.)

**Keywords:** schisandrin B, breast cancer, radiosensitivity, cell cycle, DNA double-strand break repair, gut microbiota-immune axis, short-chain fatty acids

## Abstract

**Background/Objectives**: Schisandrin B (Sch B), a bioactive lignan of *Schisandra chinensis* has been commonly investigated for its antitumor activities, yet its radiosensitizing effect and mechanism remain unclear. This study was conducted to investigate the radiosensitizing effects of Sch B in breast cancer (BC) and elucidate its molecular mechanisms, with a specific focus on the gut microbiota–immune axis. **Methods**: In vitro, CCK-8, colony formation, and 3D spheroid assays were used to evaluate the effects of Sch B on proliferation inhibition and radiosensitization, flow cytometry and immunofluorescence were used to elucidate the mechanisms involved. In vivo, 4T1 tumor-bearing mice were treated with Sch B, and 16S rDNA sequencing and LC-MS/MS were used to analyze the gut microbiota and short-chain fatty acid (SCFA) metabolism. IHC and qPCR detected antitumor immune responses. **Results**: Sch B inhibited the proliferation of BC cells in a time- and dose-dependent manner with negligible toxicity to the mammary epithelial cell line MCF-10A. Furthermore, Sch B enhanced the radiosensitivity (sensitization enhancement ratio: 1.20~1.77) of BC by inducing G1 phase cell cycle arrest and delaying radiation-induced DNA double-strand break repair. In vivo, Sch B suppressed BC growth in BALB/c mice without causing obvious systemic toxicity. Sch B reversed tumor-induced gut microbiota dysbiosis (restoring species abundance and the *Firmicutes*/*Bacteroidetes* ratio, enriching beneficial genera such as *Lactobacillus* and *Butyrobacter*) and normalized SCFA profiles (correlative evidence). Furthermore, Sch B modulated systemic immune responses by increasing the expression of *Ifng*, *Cxcl10*, *Ddx58* and promoting CD3^+^ and CD8^+^ T-cell infiltration in tumors. **Conclusions**: Sch B exerts BC radiosensitization through dual mechanisms, direct regulation of the cell cycle and DNA repair, and indirect modulation of the gut microbiota-immune axis (correlative evidence), highlighting it as a safe and effective candidate for improving the efficacy of BC radiotherapy.

## 1. Introduction

Radiotherapy (RT) is a cornerstone of breast cancer (BC) treatment, either as an adjuvant therapy for localized disease or palliative treatment for advanced tumors, by inducing DNA damage and apoptotic cell death in cancer cells [1]. However, RT is often compromised by the radioresistance of tumor cells and unavoidable toxicity to adjacent normal tissues, which restricts the achievable radiation dose and compromises treatment outcomes [2]. In light of this, the development of novel radiosensitizers that increase BC cell sensitivity to RT while sparing normal tissues is urgently needed. An ideal radiosensitizer possesses multiple favorable properties, including inherent antitumor activity, low toxicity to nor-mal cells, and the ability to potentiate the therapeutic effect of RT without adverse reac-tions. Owing to their structural diversity and relatively low toxicity, several natural prod-ucts have been investigated as promising sources of novel antitumor and radiosensitizing agents [3,4].

*Schisandra chinensis* (Turcz.) Baill., which has a long history of medicinal and dietary therapy, is commonly used in traditional immunomodulation [5]. Schisandrin B (Sch B), a bioactive lignan isolated from the fruits of *Schisandra chinensis* (Turcz.) Baill., has been extensively investigated for its various pharmacological effects, including antioxidation, inflammatory inhibition, hepatoprotective, and antiviral effects [6,7,8]. Sch B can inhibit the proliferation of multiple cancer types, such as breast cancer, lung cancer, and gallbladder cancer, by regulating cell cycle progression and inducing apoptosis [9,10,11]. Sch B has also been shown to enhance the chemotherapeutic efficacy in gastric cancer and cervical cancer [12,13]. Our previous research demonstrated that Sch B can induce G1 phase arrest in nasopharyngeal carcinoma cells by specifically targeting CDK4/6, delaying the repair of radiation-induced DNA damage, and exerting no significant inhibitory effect on normal epithelial cells, indicating that it is a promising radiosensitizer [14]. However, the potential radiosensitizing effect of Sch B in BC and its molecular mechanisms remain largely unexplored.

Research in recent years has increasingly revealed the multidimensional regulatory role that Sch B plays in host immunity. Sch B can induce pyroptosis in hepatocellular carcinoma cells via immunomodulation [15], and in asthma models, it has been shown to reduce airway eosinophil infiltration and mucus secretion by inhibiting the Th2-type immune response [16]. Sch B plays a crucial role in regulating macrophage polarization and the balance of T cell subsets [17]. There is increasing evidence that the gut microbiota–immune axis plays a pivotal role in tumor initiation and progression, as well as the response to anti-tumor therapy [18,19]. Tumor development is often accompanied by gut microbiota dysbiosis, which can suppress antitumor immunity through the alteration of short-chain fatty acid (SCFA) production and the regulation of proinflammatory or anti-inflammatory cytokine secretion [19,20,21]. Disruption of gut microbiota homeostasis has been shown to contribute to primary resistance to radiotherapy. The gut microbiota can modulate the radiation response by regulating intestinal barrier function and systemic immunity [22]. Studies have demonstrated Sch B prevents ulcerative colitis and colitis-associated cancer by activating focal adhesion kinase (FAK) and modulating the gut microbiota [23]. However, whether Sch B can regulate the gut microbiota-immune axis to enhance its antitumor effects and synergistically promote radiosensitization remains unknown.

We hypothesized that Sch B serves as a potent radiosensitizer for BC by targeting cell cycle progression and DNA damage repair, while simultaneously associatingthe gut microbiota-immune axis to enhance antitumor immunity. To test this hypothesis, we evaluated the anti-tumor and radiosensitizing effects of Sch B. The molecular mechanisms of its radiosensitizing activity, with a special focus on cell cycle arrest and DNA damage repair processes was investigated. We also studied the impacts of Sch B on the gut microbiota composition, SCFA metabolism, systemic immune responses, and tumor-infiltrating immune cell profiles. Our findings seek to provide comprehensive evidence validating Sch B as a promising candidate with multiple synergistic antitumor mechanisms for BC radiotherapy.

## 2. Results

### 2.1. Sch B Inhibits BC Cell Proliferation In Vitro and Suppresses Tumor Growth In Vivo

**Figure 1:**
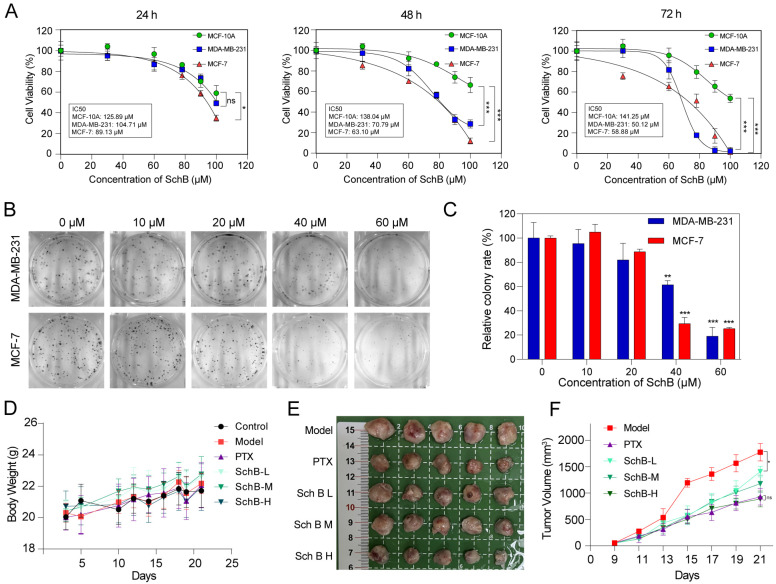
Sch B inhibits BC cell proliferation in vitro and suppresses tumor growth in vivo. (**A**) Viability of BC cells (MBA-MD-231 and MCF-7) and mammary gland epithelial cells MCF-10A treated with Sch B at concentrations of 0, 20, 40, 60, 80, and 100 μM for 24, 48, and 72 h (*n* = 5 per group). (**B**) Representative images of colony formation assays for BC cells treated with the indicated concentrations of Sch B (*n* = 3 per group). (**C**) Quantitative analysis of the relative colony formation rate. Colony numbers were normalized to those in the 0 μM (vehicle control) group. (**D**) Body weight curves of mice bearing 4T1 subcutaneous tumors were measured at different time points after treatment with PTX and Sch B at different concentrations (*n* = 5 per group). (**E**) Representative images of dissected tumors from the Model, PTX and Sch B treatment groups at the endpoint. (**F**) Tumor volume growth curves measured every 2 days. All data are presented as mean ± SD. * *p* < 0.05, ** *p* < 0.01, *** *p* < 0.001.

Given that the anti-tumor mechanisms of Sch B are predominantly centered on inhibiting cell proliferation, we initially evaluated its effects on the proliferation of BC cells and mammary epithelial cell line MCF-10A. The CCK-8 results revealed that Sch B inhibited the proliferation of BC cell lines. As the duration of Sch B treatment and its concentration increased, the proliferation inhibition of MDA-MB-231 cells was gradually enhanced, with calculated IC50 values of 104.71, 70.79, and 50.12 μM at 24, 48, and 72 h, respectively (Figure 1A). Sch B exerted a similar inhibitory effect on another BC cell line MCF-7, with IC50 values of 89.13, 63.10, and 58.88 μM at the same corresponding time points. However, Sch B exhibited selective toxicity, sparing mammary epithelial cell MCF-10A, with negligible cytotoxicity observed at concentrations ≤ 60 μM (Figure 1A). The colony formation assay also demonstrated that Sch B exerted an inhibitory effect on BC cell lines (Figure 1B,C), and the clonogenic capacity of BC cells was impaired. These findings indicate that Sch B suppresses BC cell proliferation while showing favorable safety profiles against normal mammary gland epithelial cells.

To further validate these in vitro observations in a physiological context, a murine subcutaneous tumor model was established using 4T1 BC cells. After tumor formation, the mice were treated with paclitaxel (PTX, 100 mg/kg) or Sch B at low (20 mg/kg), medium (40 mg/kg), or high (80 mg/kg) doses for 14 consecutive days (Figure S1A). No differences in body weight were detected across the experimental groups throughout the treatment period. However, mice in the Sch B-treated groups showed slightly higher body weights, reflecting the favorable in vivo safety profile of Sch B (Figure 1D). Relative to the model group, the tumor volumes were significantly decreased in mice treated with PTX or Sch B at all tested doses. The anti-tumor efficacy of high-dose Sch B was comparable to that of PTX (a first-line chemotherapeutic agent for BC), confirming that Sch B exerts a potent anti-tumor effect in vivo (Figure 1E,F).

### 2.2. Sch B Enhances the Radiosensitivity of BC Cells

**Figure 2:**
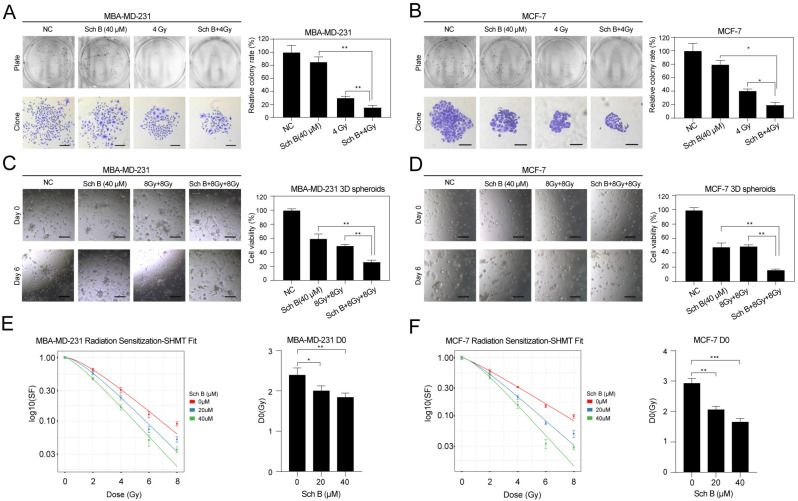
Sch B enhances the radiosensitivity of BC cells. (**A**,**B**) Colony formation assay of MDA-MB-231 and MCF-7 cells treated with Sch B (40 μM), radiation (4 Gy), or the combination of Sch B and radiation (40 μM + 4 Gy). (**C**,**D**) Bright-field images of MDA-MB-231 and MCF-7 3D spheroids on day 0 and day 6 after treatment with Sch B (40 μM), radiation (8 Gy + 8 Gy), or Sch B combined with radiation (40 μM + 8 Gy +8 Gy) (Scale bar = 200 μm). Cell viability of MDA-MB-231 and MCF-7 3D spheroids was assessed on day 6. (**E**,**F**) Cell survival curves of MDA-MB-231 and MCF-7 cells fitted using the Single-Hit Multi-Target model under adiation doses from 0 Gy to 8 Gy. The radiobiological parameter D0, which was used to calculate the SER, was derived from these curves. Data are presented as mean ± SD (*n* = 3). * *p* < 0.05, ** *p* < 0.01, *** *p* < 0.001.

Ideally, radiosensitizers should exert no significant cytotoxicity at their working concentrations. When selecting the concentration of Sch B for combination with X-ray radiation in BC cells, we used the 24 h IC50 values for Sch B as determined by the CCK-8 assay: 104.71 μM for MDA-MB-231 cells and 89.13 μM for MCF-7 cells. At a concentration of 40 μM, the cell viability of both BC cell lines exceeded 95% (Figure 1A). Thus, BC cells were pretreated with 40 μM Sch B for 6 h followed by X-ray radiation, and the radiosensitizing effect of Sch B was evaluated. In BC cells, the colony formation rate in the Sch B-combined radiation group was significantly lower than that in the radiation group (Figure 2A,B). These results indicate that Sch B enhances the radiosensitivity of BC. Tumor cells exhibit phenotypic differences between two-dimensional (2D) and three-dimensional (3D) culture systems due to variations in the microenvironment. To strengthen the evidence for the radiosensitizing effect of Sch B, we further verified its effects in 3D spheroid models derived from BC cells. Consistent with the 2D culture results, 3D spheroid viability was significantly reduced in the Sch B-combined radiation group (Sch B + 8 Gy + 8 Gy) compared with that in the radiation group (8 Gy + 8 Gy) in both BC 3D spheroid models (Figure 2C,D) (drug and RT administration schedule refers to Figure S1B).

In addition, we fitted the cell survival curves of Sch B at 0 μM, 20 μM, and 40 μM under radiation doses from 0 Gy to 8 Gy using the colony formation assay and single-hit multitarget model, and derived the mean lethal dose (D0) of BC cells at each Sch B concentration (Figure 2E,F). SER = (D0 of the radiation group)/(D0 of the Sch B-combined radiation groups). For MDA-MB-231 cells, the SER values of Sch B at 20 μM and 40 μM were 1.20 and 1.30, respectively. For MCF-7 cells, the corresponding SER values were 1.42 and 1.77. Sch B exerted stable radiosensitization from low doses (2 Gy) to high doses (8 Gy). Generally, the radiosensitizing efficacy of a drug is interpreted based on the range of SER values. When SER ≥ 1.0, the drug is concerned to enhance radiotherapy efficacy. Therefore, integrating multiple lines of evidence, including the findings from 2D and 3D tumor models as well as the SER values, we confirmed that Sch B exerts a definite radiosensitizing effect on BC cells.

### 2.3. Sch B Achieves Radiosensitizing Effect by Arresting BC Cells at the G1 Phase and Delaying DNA Damage Repair

**Figure 3:**
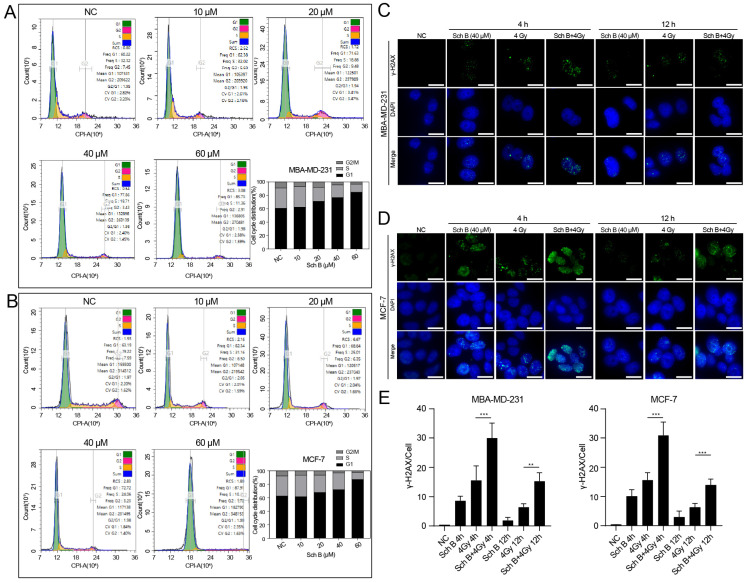
Sch B achieves radiosensitization by arresting cells at the G1 phase and delaying DNA damage repair. (**A**,**B**) Flow cytometric analysis of cell cycle distribution in MDA-MB-231 and MCF-7 cells treated with Sch B at concentrations of 0 (vehicle control), 10, 20, 40, and 60 μM. (**C**,**D**) Effects of Sch B, radiation, and their combination on γ-H2AX foci formation in MDA-MB-231 and MCF-7 cells. Representative images of cell nuclei in each group are shown (scale bar = 20 μm). (**E**) Histograms showing the number of γ-H2AX foci per nucleus. Data are presented as mean ± SD (*n* = 3), ** *p* < 0.01, *** *p* < 0.001.

There are three well-established mechanisms underlying the action of radiosensitizers that have been identified: arresting the cell cycle at phases sensitive to radiotherapy, delaying DNA damage repair, and activating or inhibiting signaling pathways associated with radiosensitivity [24]. Considering this, we first investigated the effect of Sch B on the cell cycle distribution of BC cells using flow cytometric analysis. As the Sch B concentration increased (10–60 μM), there was a notable increase in the percentage of MDA-MB-231 and MCF-7 cells arrested in the G1 phase (Figure 3A,B). Double-strand breaks (DSBs) are the primary form of DNA damage induced by ionizing radiation, and enhanced DSB repair capacity is a critical mechanism underlying tumor cell radioresistance in radiotherapy. We utilized immunofluorescence staining of γ-H2AX (DSB marker) to quantify DNA damage in BC cell lines following treatment with Sch B, radiation, or their combination. Figure 3C–E illustrates that, 4 h following 4 Gy radiation, the Sch B + 4 Gy group exhibited γ-H2AX foci per nucleus of 30.0 in MDA-MB-231 and 30.9 in MCF-7, markedly exceeding the 4 Gy only group, which showed 15.6 for MDA-MB-231 and 15.7 for MCF-7, suggesting that Sch B boosts the DNA damage resulting from radiation. At 12 h after irradiation, γ-H2AX foci in the 4 Gy group were markedly diminished relative to those at 4 h post-irradiation (4 Gy, 4 h), indicating efficient repair of radiation-induced DNA damage. In sharp contrast, the number of γ-H2AX foci in the Sch B + 4 Gy group at 12 h (15.3 and 13.9 per nucleus) remained comparable to that in the 4 Gy group at 4 h (15.6 and 15.7 per nucleus). These findings demonstrated that Sch B delays the repair of radiation-induced DNA damage in breast cancer cells, which constitutes a critical mechanism underlying its radiosensitizing activity.

### 2.4. Sch B Elicits Host Immune Competence to Trigger Antitumor Immune Responses

**Figure 4:**
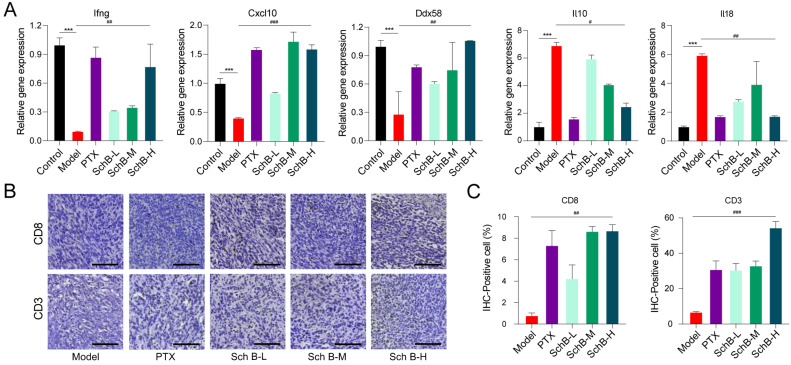
Sch B mobilizes host immunity to initiate anti-tumor immune response. Serum and tumor samples were collected from 4T1 tumor-bearing mice. Total RNA was isolated from serum for qPCR analysis, while tumor tissues were subjected to IHC staining. (**A**) Effects of Sch B treatment on serum levels of *Ifng*, *Cxcl10*, *Ddx58*, *Il10* and *Il18* in mice. (**B**) IHC staining of CD8 and CD3 in tumor tissues from PTX and Sch B at different concentration groups. Representative staining images are presented (*n* = 3, Scale bar = 100 μm). (**C**) The number of IHC-positive cells was quantified in six microscopic fields per sample (*n* = 6 fields). *** *p* < 0.001 vs. control group; ^#^ *p* < 0.05, ^##^ *p* < 0.01, ^###^ *p* < 0.001 vs. model group.

Accumulating evidence has demonstrated that Sch B can induce pyroptosis in hepatocellular carcinoma cells via activating immune cells [15], suggesting a potential immunomodulatory role of Sch B. Accordingly, we hypothesized that Sch B might also exert anti-tumor effects by mobilizing host immune responses in BC. To validate this, we further performed in vivo animal experiments and firstly detected the relative expression levels of immune-related indicators—*Ifng*, *Cxcl10*, *Ddx58*, *Il10*, and *Il18* in the blood of tumor-bearing mice treated with PTX or Sch B at different concentrations using qPCR. The mRNA levels of *Ifng*, *Cxcl10*, and *Ddx58*, which activate T cells and inhibit tumors, were significantly decreased in the serum of tumor-bearing model mice. In contrast, the mRNA levels of *Il10* and *Il18*, which exert immunosuppressive effects and facilitate tumor cell immune escape, were significantly elevated. Following treatment with paclitaxel (PTX) or Sch B at various concentrations, the mRNA levels of *Ifng*, *Cxcl10*, and *Ddx58* were significantly increased, while those of *Il10* and *Il18* were reduced in the treatment groups. Notably, the ability of the high-dose Sch B group to restore or inhibit these indicators was comparable to or even superior to that of the PTX group (Figure 4A). The expression of CD8 and CD3 in tumor tissues from each group was detected by IHC staining. Consistent with previous reports indicating that CD8^+^ T cells are critical for IFN-mediated antitumor effects [25], we found that treatment with PTX or Sch B at different concentrations promoted CD8^+^ T cell infiltration. The proportions of CD3^+^ and CD8^+^ positive cells in tumors were significantly increased in all Sch B treatment groups, in which *Ifng* levels in the blood were elevated (Figure 4B,C). These results indicate that tumors induce immunosuppression by modulating multiple host immune-regulating factors to achieve immune escape. The treatment of Sch B at various doses mobilizes the host antitumor immune response, partially restores the levels of a cascade of immune factors in the blood, and enhances the immune infiltration of CD8^+^-T cells in tumors. However, the activation of anti-tumor immunity mediated by Sch B in this study was only inferred from the elevated levels of immune-stimulatory factors and increased T-cell infiltration. Further functional verification is therefore required in subsequent research.

### 2.5. Effects of Sch B on Gut Microbiota Diversity and Community Species Composition in Tumor-Bearing Mice

**Figure 5:**
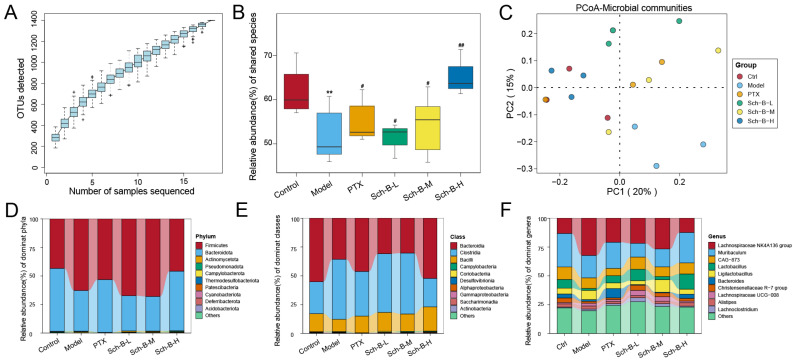
Effects of Sch B on gut microbiota diversity and community species composition in tumor-bearing mice. (**A**) Species accumulation curve of 18 samples (OTU: operational taxonomic unit, used to define bacterial taxa at 97% sequence similarity). (**B**). Relative abundance of shared species. (**C**) PCoA analysis among control, model, Sch-B-L, Sch-B-M, Sch-B-H groups at microbial communities. (**D**–**F**) Classification levels of phylum, class, and genus of gut microbiota in mice. Data are presented as mean ± SD (*n* = 3), ** *p* < 0.01 vs. control group; ^#^ *p* < 0.05, ^##^ *p* < 0.01, vs. model group.

The gut microbiota-immune axis is recognized as a key bridge in tumor regulation, and several traditional Chinese medicine (TCM) components or their active ingredients have been shown to exert therapeutic effects by modulating the gut microbiota [26]. After confirming that Sch B can mobilize host immunity, we performed 16S rDNA sequencing on the mice’s intestinal contents. The rarefaction curves of the 18 intestinal content samples plateaued, suggesting that the sequencing depth was sufficient to cover most species for subsequent analyses (Figure 5A). Analysis of gut microbiota species abundance revealed that the tumor-bearing model group exhibited a significant reduction in gut microbiota abundance compared with the untreated control group. In contrast, PTX (paclitaxel, 100 mg/kg) or low (20 mg/kg)-, medium (40 mg/kg)-, and high (80 mg/kg)-dose Sch B showed significantly increased gut microbiota abundance (Figure 5B). This indicates that the tumor-induced abundance decrease was partially restored after treatment with PTX or Sch B. Notably, the gut microbiota abundance in the high-dose Sch B group was higher than that in the PTX group, suggesting that Sch B exerted a superior effect to the chemotherapeutic drug PTX in maintaining gut microbiota homeostasis. PCoA score plots showed that the gut microbiota composition of the tumor-bearing model group clustered separately in one quadrant, distinct from the other groups. The gut microbiota composition of the high-dose Sch B group was highly similar to that of the control group (Figure 5C).

At the phylum level, *Firmicutes* and *Bacteroidetes* represented the two predominant phyla in the gut microbiota across all experimental groups, accounting for the majority of microbial community abundance. The tumor-bearing model group exhibited a markedly elevated Firmicutes/Bacteroidetes ratio (F/B), which is a key indicator reflecting gut microbiota dysbiosis. The F/B was reduced in both the PTX treatment group and the Sch B treatment groups (at low, medium, and high concentrations), suggesting a regulatory effect of these agents on gut microbial phylum-level homeostasis (Figure 5D). At the class level, *Bacteroidia*, *Clostridia*, and *Bacilli* were the predominant classes across all groups. The model group exhibited a higher abundance of *Clostridia* and a lower abundance of *Bacteroidia* and *Bacilli*. After drug treatment, the abundances of these three classes tended to revert to those in the control group (Figure 5E). Similarly, the composition at the genus level also showed a trend toward that of the normal control group following drug intervention (Figure 5F). Overall, Sch B intervention ameliorated the tumor-induced alterations in gut microbiota, which tended to resemble the gut microbiota profile of the control group mice.

### 2.6. Sch B Modulates Host Immunity via the Gut Microbiota-Immune Axis

**Figure 6:**
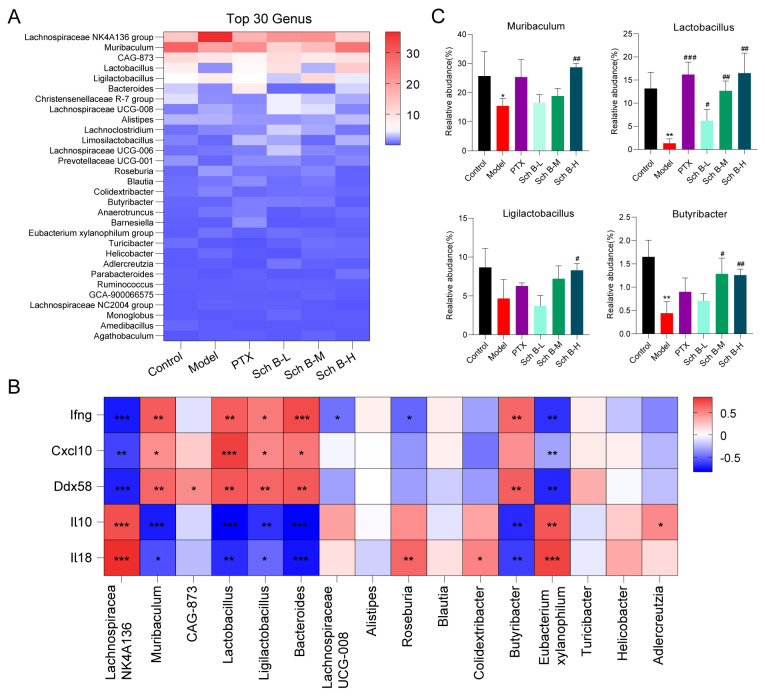
Sch B correlated with host immune modulation via the gut microbiota-immune axis (correlative analysis). (**A**) Correlation analysis between gut microbial genera and immunity-related biochemical indicators. Red cells indicate a positive correlation (*r* > 0), while blue cells indicate a negative correlation (*r* < 0). (**B**) Differential analysis of the relative abundances of *Muribaculum*, *Lactobacillus*, *Ligilactobacillus*, and *Butyribacter*, which were associated with tumorigenesis or tumor progression, among the six groups. Data were expressed as means ± SEM (*n* = 3). * *p* < 0.05, ** *p* < 0.01, *** *p* < 0.001 vs. control group; ^#^ *p* < 0.05, ^##^ *p* < 0.01 vs. model group. (**C**) Differential analysis of the relative abundances of *Muribaculum*, *Lactobacillus*, *Ligilactobacillus*, and *Butyribacter*, which were associated with tumorigenesis or tumor progression, among the six groups.

To explore the potential mechanism through which Sch B modulates host immunity, we further analyzed the gut microbiota composition at the genus level and its correlation with immunity-related indicators. At the genus taxonomic level, the thirty most abundant microbial genera across all samples were visualized via hierarchical clustering heatmap to intuitively reflect their relative abundance patterns (Figure 6A). The associations between the abundances of these top 30 genera and the immunity-related indicators *Ifng*, *Cxcl10*, *Ddx58*, *Il10*, and *Il18* were analyzed, which uncovered distinct correlative patterns between specific microbial genera and host immune status (Figure 6B). The results demonstrated a significant positive correlation (correlation coefficient *r* > 0) between the relative abundances of several beneficial genera, including *Muribaculum*, *CAG-873*, *Lactobacillus*, *Ligilactobacillus*, *Bacteroides*, and *Butyribacter,* and the levels of *Ifng*, *Cxcl10*, and *Ddx58*, while these genera exhibited a significant negative correlation with *Il10* and *Il18*. Conversely, the relative abundances of *Lachnospiraceae_NK4A136*, *Eubacterium xylanophilum*, *Lachnospiraceae UCG-008*, and *Roseburia* were remarkably negatively correlated (*r* < 0) with *Ifng*, *Cxcl10*, and *Ddx58* levels, but positively correlated with *Il10* and *Il18* levels.

Further analysis identified significant intergroup differences in the relative abundances of *Muribaculum*, *Lactobacillus*, *Ligilactobacillus*, and *Butyribacter*, which are genera closely associated with tumorigenesis and progression. As shown in Figure 6B, compared with normal mice, the relative abundances of *Muribaculum*, *Lactobacillus*, and *Butyribacter* in the intestinal contents of tumor-bearing mice were drastically decreased. These abundances were significantly increased following Sch B treatment. These findings demonstrate that Sch B correlates with host immune modulation via the gut microbiota-immune axis (correlative analysis).

### 2.7. Effects of Sch B on SCFA Production

**Figure 7:**
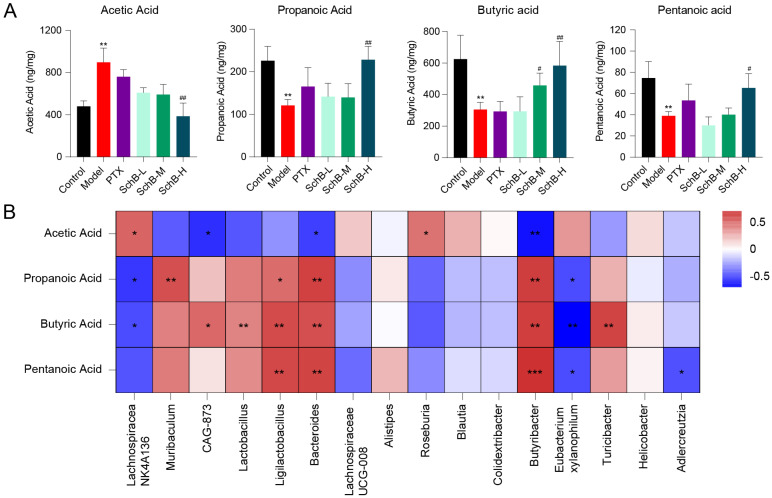
Effects of Sch B on SCFA production. (**A**) Difference analysis of the relative abundance of acetic acid, propanoic, butyric, and pentanoic acid. (**B**) Correlation analysis between gut microbial genera and four SCFAs. Red cells represent positive correlation (*r* > 0), and blue cells represent negative correlation (*r* < 0). Data were expressed as means ± SEM (*n* = 5). * *p* < 0.05, ** *p* < 0.01, *** *p* < 0.001 vs. control group; ^#^ *p* < 0.05, ^##^ *p* < 0.01 vs. model group.

SCFAs are mainly produced through the anaerobic fermentation of dietary fiber by gut microbiota. Their levels can reflect the homeostasis of gut microbiota, and abnormal levels are associated with host infection or tumorigenesis. SCFAs encompass acetic acid, propionic acid, butyric acid, and pentanoic acid, which serve as the key metabolites of the gut microbiota [27,28]. To assess the effect of Sch B on gut microbiota-derived SCFA production, the concentrations of the aforementioned SCFAs in mouse cecal contents were quantified via LC-MS/MS. As shown in Figure 7A, the tumor-bearing model group showed a significant decrease in propionic, butyric, and pentanoic acid, accompanied by a notable elevation in acetic acid concentrations (*p* < 0.01). Following treatment with Sch B, the Sch B-H group showed modulated SCFA profiles, with significant downregulation of acetic acid and upregulation of propionic, butyric, and pentanoic acid to varying degrees. PTX treatment failed to exert a significant ameliorative effect on SCFA imbalance.

The potential correlations between SCFAs and the top 15 most abundant microbial genera were assessed (Figure 7B). The content of acetic acid exhibited a significant positive correlation with the relative abundances of *Lachnospiraceae_NK4A136* and *Roseburia* (*r* > 0), negatively correlated with those of *CAG-873*, *Bacteroides*, and *Butyribacter* (*r* < 0). *Butyric acid* content exhibited a significant positive correlation with several genera, including *CAG-873*, *Lactobacillus*, *Ligilactobacillus*, *Bacteroides*, *Butyribacter*, and *Turicibacter*. Propionic acid and pentanoic acid contents were significantly correlated with genera such as *CAG-873*, *Lactobacillus*, and *Ligilactobacillus* (positive correlation). These results demonstrate that Sch B correlates with the reversal of gut microbiota dysbiosis triggered by tumor development and the normalization of SCFA production synthesized by the microbial community.

## 3. Discussion

RT remains a primary therapeutic modality for BC; however, its efficacy is severely hampered by tumor radioresistance and off-target toxicity to normal tissues [2]. The development of ideal radiosensitizers that can potentiate RT efficacy while sparing normal cells has long been a critical need in clinical oncology. Despite decades of research, clinically approved radiosensitizers, such as cisplatin and gemcitabine, are limited by inherent drawbacks that restrict their application [29,30]. First, most synthetic radiosensitizers exhibit non-selective cytotoxicity, causing severe adverse reactions, such as myelosuppression, gastrointestinal toxicity, and organ damage, which often force dose reduction or treatment interruption [3,31]. Second, their mechanisms of action are typically monodimensional, lacking regulation of other radiotherapy resistance pathways and thereby rendering tumors prone to developing acquired resistance through the activation of compensatory pathways [32,33]. Third, except for nanomaterial-based radiosensitizers [34,35], few conventional radiosensitizers can modulate the host immune microenvironment and synergize with RT-induced anti-tumor immunity.

Our findings indicate that Sch B overcomes these limitations by integrating multiple favorable properties of an ideal radiosensitizer. Sch B is a lipophilic lignan with favorable oral bioavailability in rodents. Previous pharmacokinetic studies indicated that oral Sch B (40 mg/kg) presents a Tmax of 1.00 h and a t_1/2_ of 9.40 h. This compound predominantly accumulates in the liver, with renal excretion as the major route of elimination [36]. The mouse doses used in this study (20–80 mg/kg) translate to 1.6–6.4 mg/kg in humans by body-surface-area conversion. Sch B shows low systemic toxicity and favorable tissue distribution, supporting its clinical potential as a radiotherapy adjuvant. However, formal human pharmacokinetics, optimal administration routes, and combined radiotherapy schedules remain to be validated in clinical trials. Our results demonstrate that Sch B possesses dual direct radiosensitizing mechanisms and unique gut microbiota-mediated immunomodulatory effects in BC. As a key prerequisite for clinical translation, Sch B inhibited the proliferation of BC cell lines in a time- and dose-dependent manner (IC50: 50.12~70.79 μM at 72 h) while exerting negligible toxicity to normal MCF-10A cells (Figure 1A), and in vivo treatment (20~80 mg/kg) did not cause obvious body weight loss and even showed a slight protective effect on general status compared to the tumor-bearing model mice (Figure 1D) . Mechanistically, Sch B arrested BC cells in G1 phase and delayed RT-induced DNA DSB repair (Figure 3A–E), with an SER of 1.20–1.77 validating its efficacy. More importantly, unlike conventional radiosensitizers that exert single or limited radiosensitizing mechanisms, we have demonstrated that Sch B not only acts as a radiosensitizer but also associates with reversing tumor-induced gut microbiota dysbiosis. This reversal is correlated with enhanced systemic immunity, synergizing with RT to inhibit tumor growth.

Tumor cell radiosensitivity differs across distinct cell cycle phases. In general, cells in the G2/M phase exhibit the highest radiosensitivity, followed by those in the G1 phase. Cells entering the S phase typically exhibit obvious radioresistance [14,37]. Although G1 phase arrest does not universally enhance tumor radiosensitivity, the radiosensitizing effect of Sch B is not solely attributable to G1 arrest. Instead, the dominant mechanism involves impaired repair of radiation-induced DSBs, as shown by persistent γ-H2AX foci. Sch B traps cells in G1, a phase with relatively low DSB repair proficiency, and directly delays DNA damage repair. This dual action cooperatively enhances radiation-induced lethality. In addition, p53 is a pivotal tumor suppressor that triggers G1 checkpoint arrest mainly by transcriptionally inducing p21, which inhibits CDK4/6-cyclin D1 complexes and blocks G1/S transition [38]. According to the published study, Sch B significantly upregulates p53 and p21 expression, and downregulates CDK4, CDK6, and cyclin D1, thereby inducing obvious G0/G1 phase arrest in lung adenocarcinoma [10]. The G1 phase arrest induced by Sch B in BC in the current study is most likely mediated through the activation of p53 signaling.

Radiosensitization includes two mechanisms: directly enhancing tumor sensitivity to radiation and indirectly improving anti-tumor efficacy [39]. Our in vitro data confirmed that Sch B directly promotes radiosensitivity by regulating cell cycle arrest and DNA damage repair in BC cells. Meanwhile, our in vivo results revealed that Sch B enhances the anti-tumor effect of radiotherapy by remodeling gut microbiota and boosting anti-tumor immunity. These two parts are complementary rather than independent, and together constitute the dual mechanism of Sch B-mediated radiosensitization, which is the major novelty of this study. The gut microbiota are key regulators of tumor initiation, progression, and response to anti-tumor therapies [18,19]. They modulate tumor immunity primarily through metabolic products, SCFAs, and the regulation of cytokine secretion [20,40]. Regarding radiotherapy, gut microbiota dysbiosis has been associated with increased radioresistance by impairing intestinal barrier function and suppressing anti-tumor T cell responses, while restoring gut homeostasis can enhance RT efficacy [22]. Gut microbiota can be functionally classified into tumor-promoting and tumor-suppressing groups based on their roles in tumor biology. Tumor-suppressing genera such as *Lactobacillus*, *Bacteroides* and *Butyribacter* are known to produce SCFAs that enhance intestinal barrier integrity, promote T cell differentiation, and inhibit pro-inflammatory cytokine production. Tumor-promoting genera like *Lachnospiraceae_NK4A136* and *Roseburia* often disrupt gut homeostasis, induce chronic inflammation, and suppress anti-tumor immunity [27]. Consistent with the aforementioned findings, tumor-bearing mice in our study exhibited reduced abundances of *Muribaculum*, *Lactobacillus*, and *Butyribacter* (tumor-suppressing genera) and increased levels of *Lachnospiraceae_NK4A136* and *Eubacterium xylanophilum* (tumor-promoting genera) (Figure 6B). Sch B treatment significantly reversed these changes, suggesting that its immunomodulatory effects are at least partially mediated by reshaping the gut microbiota composition. SCFAs, the main metabolites of gut microbiota fermentation, play a pivotal role in linking gut microbiota to systemic immunity [41,42]. Butyrate and propionate, in particular, have been proven to enhance CD8+ T cell activation and infiltration in tumors, while reducing the function of immunosuppressive cells [43,44]. Our LC-MS/MS results showed that tumor-bearing mice had decreased levels of propionate, butyrate, and pentanoic acid, and increased acetic acid (Figure 7A), which is consistent with previous studies that tumor-associated gut dysbiosis disrupts SCFA metabolism [27]. Sch B treatment normalized these SCFA profiles, with high-dose Sch B significantly increasing propionate and butyrate levels (Figure 7A). Spearman correlation analysis further confirmed that the relative abundances of beneficial genera (e.g., *Lactobacillus*, *Butyribacter*) were positively correlated with *Ifng*-, *Cxcl10*, and *Ddx58*- levels, and negatively correlated with *Il10* and *Il18* (Figure 6B). These results support a coordinated immune–metabolic axis associated with Sch B treatment, in which gut microbiota remodeling and SCFA restoration correlate with enhanced systemic immunity and intratumoral T-cell infiltration, potentially complementing its direct radiosensitizing effects. This mechanism is consistent with the regulatory pattern of natural products reported in recent studies [45], highlighting the potential of Sch B as a “multi-dimensional” agent that integrates direct tumor cell killing, radiosensitization, and gut microbiota-mediated. However, the immune analysis of this study is limited to the detection of immune factor mRNA and CD3^+^/CD8^+^ T cell infiltration, lacking the analysis of immune cell subsets and functional verification. The observed immune changes are phenotypic rather than functional.

A PTX control group was incorporated into the animal experiments due to the fact that PTX is frequently combined with radiotherapy for tumor treatment in clinical practice, where it exerts a radiosensitizing effect. Initially, we hypothesized that although PTX treatment could inhibit tumor growth, it would induce gut microbiota dysbiosis in mice and might exhibit partial immunosuppressive effects. This is because chemotherapy-induced constipation and diarrhea are common clinical side effects [46], and paclitaxel-based chemotherapy is also frequently associated with reduced immunity and neutropenia [47]. However, our results showed that PTX administration increased the levels of immunity-enhancing factors (*Ifng*, *Cxcl10*, and *Ddx58*) in the blood and partially restored tumor-induced gut microbiota dysbiosis, which seemed inconsistent with our initial expectations. Literature evidence indicates that low concentrations of PTX can enhance anti-tumor immune responses, and chemotherapy induces temporary immune reconstitution and augments anti-tumor immune responses, with the 12–14 day window period after chemotherapy potentially representing the optimal time frame [48]. In the present study, to match the concentration of Sch B, we selected a PTX administration regimen of 100 mg/kg every 4 days, resulting in only three administrations over the 2-week treatment period. Thus, the elevated levels of immunity-related factors in the blood, enhanced immune infiltration into tumors, and improved gut microbiota profiles observed in the PTX-treated group are consistent with previous reports that low-concentration and short-term (12–14 days) PTX administration can enhance host immunity. In contrast, a key advantage of Sch B is that its capacity to enhance host immunity is not restricted to low concentration ranges. Both the present study and several previous animal experiments have demonstrated that Sch B exerts no significant toxicity to normal tissues and organs, even at relatively high concentrations, and may even confer certain protective effects [7,49]. The present study evaluated only short-term (14-day) efficacy and safety. Long-term efficacy, potential acquired drug resistance, and chronic toxicity were not assessed. Future studies will extend treatment to ≥4 weeks to monitor tumor regrowth, resistance development, chronic organ toxicity, body-weight stability, and survival duration. Previous reports indicate that long-term Sch B administration (0.012% (*w*/*w*) of diet for 84 weeks does not induce obvious organ (brain, heart, liver, and kidney) damage in mice but retards the aging process, supporting favorable long-term safety [50].

This study makes several notable contributions to the field of radiosensitizer development and natural product research. First, we systematically demonstrate that Sch B radiosensitizes BC through dual direct mechanisms (G1 phase arrest and delayed DNA damage repair) and indirect regulation of the gut microbiota-immune axis, providing a novel multidimensional therapeutic paradigm for BC radiotherapy. Second, we validated that Sch B has a favorable safety profile with minimal toxicity to normal cells, addressing the key limitation of current synthetic radiosensitizers. The ability of Sch B to modulate the gut microbiota-immune axis also suggests potential synergistic effects with immunotherapies, which warrants further exploration in combination therapy studies. Additionally, the correlation between specific gut microbial genera, such as *Lactobacillus* and *Lachnospiraceae_NK4A136*, and the treatment response may indicate potential biomarkers for predicting the efficacy of Sch B-based combination therapy.

Despite the comprehensive findings, limitations also should be acknowledged. First, no in vivo radiotherapy model was used to confirm the therapeutic effect of Sch B combined with radiation, and the conclusion of in vivo radiosensitization is based on an in vitro surrogate assay. Second, the causal relationship between Sch B-mediated gut microbiota remodeling and enhanced anti-tumor immunity/radiosensitization remains to be verified. The observed changes in microbiota, SCFAs, and immune factors may be parallel events or sequential regulatory processes. Third, although we have separately validated Sch B’s direct radiosensitizing effects and its indirect anti-tumor activity via modulation of the gut microbiota immune axis, the causal and synergistic relationships between these two core mechanisms remain unelucidated. In subsequent studies, we will perform FMT from Sch B-treated tumor-bearing mice to antibiotic-induced microbiota-depleted mice, combined with in vivo radiotherapy models, to directly verify whether the gut microbiota–immune axis is a necessary mediator of Sch B-induced radiosensitization. In addition, gut microbiota can be affected by diet, housing environment, and circadian rhythm. Although all mice were kept under standardized SPF conditions, potential external interference cannot be completely excluded.

## 4. Materials and Methods

### 4.1. Cell Culture

BC cell lines MDA-MB-231, MCF-7, 4T1 and mammary epithelial cell line MCF-10A were kindly provided by the Cell Bank of the Chinese Academy of Sciences (Beijing, China). MDA-MB-231, MCF-7 and 4T1 cells were cultured in DMEM/F12 medium (Gibco, Grand Island, NY, USA, 11320033) supplemented with 10% fetal bovine serum (FBS, PAN Seratech, Aidenbach, Germany, ST30-3302). MCF-10A were maintained in DMEM/F12 supplemented 10% FBS and 100 ng/mL cholera toxin (MCE, Monmouth Junction, NJ, USA, HY-P1446), 10 μg/mL insulin (PeproTech, Cranbury, NJ, USA, 10-365), and 20 ng/mL EGF (Peprotech, AF-100-15). All the cell lines were incubated in 5% CO_2_, 37 °C.

### 4.2. CCK-8 Cytotoxicity-Proliferation Assay

Cells were seeded into 96-well plates at a density of 3000 cells per well. After overnight incubation to facilitate cell adhesion, they were treated with 20~100 μM Sch B (Selleck, Houston, TX, USA, S3600) and cultured for 24 h, 48 h, and 72 h, respectively. Cells were incubated with a mixture of 100 μL DMEM/F12 medium and 10 μL of CCK-8 reagent at 37 °C for 2 h. The optical density (OD) values were measured via a microplate reader (Bio-Rad Laboratories, Hercules, CA, USA).

### 4.3. Colony Formation Assay

#### 4.3.1. Inhibition Effect of Sch B on BC Cell Proliferation

Cells were seeded in 6-well plates at a density of 800 cells per well. After being treated with Sch B for 48 h, the medium containing Sch B was replaced with a medium without Sch B. After 7~10 d of culturing, the colonies were fixed and stained. Colony images were captured using a gel imaging system (Bio-Rad, Hercules, CA, USA), and colonies containing ≥ 50 cells were counted manually.

#### 4.3.2. Radiosensitivity Amplifying Effect of Sch B on BC Cells

Cells was divided into control group (0 μM Sch B + 0 Gy RT), Sch B monotherapy group (40 μM Sch B + 0 Gy RT), RT monotherapy group (0 μM Sch B + 4 Gy RT) and Sch B-combined RT group (40 μM Sch B + 4 Gy RT). For the Sch B-pretreated groups (Sch B monotherapy and Sch B-combined RT group), the cells were incubated with Sch B for 6 h. The RT monotherapy and Sch B-combined RT groups were exposed to RT at a dose of 4 Gy, using a medical linear accelerator (6 MV energy, 600 cGy/min dose rate, 100 cm distance) under atmospheric conditions. After radiation, the culture medium was refreshed to remove residual Sch B, and the cells were cultured for 7~10 d under standard conditions. The colonies were fixed with 4% paraformaldehyde, stained with 0.1% crystal solution. Subsequently, photographs were taken.

#### 4.3.3. Sensitization Enhancement Ratio (SER) of Sch B on BC Cells

The experiment was divided into a radiation group (0~8 Gy) and Sch B-combined RT groups, with the latter receiving the same radiation doses as the radiation group. Cells were trypsinized and seeded into 6-well plates 500 cells per well for the 0~6 Gy groups, 800 cells per well for the 8 Gy group. After cell attachment, the medium in the Sch B-combined RT groups was replaced with medium containing Sch B 20 μM and 40 μM cultured for 6 h prior to radiation exposure. Before radiation, the medium in all experimental groups was replaced with normal medium, and cultured for 10 d, then fixed and stained. Cloning efficiency (Plating Efficiency, PE) was calculated by counting colonies containing more than 50 cells, with the formula PE = number of colonies/number of seeded cells. Surviving fraction (SF) = PE of irradiated cells/PE of 0 Gy-irradiated cells. Cell survival curves after exposure to 0~8 Gy were fitted using GraphPad Prism software 8.4.3 with the multitarget single-hit model SF = 1 − (1 − e^−D/D0^)^N^. Radiological parameters including N and D0 were calculated, and the sensitization enhancement ratio (SER) was determined as SER = D0 of the radiation group/D0 of the Sch B-combined radiation groups. D refers to the radiation dose, D0 denotes the mean lethal dose, and N represents the number of radiation-sensitive targets in cells.

### 4.4. Three-Dimensional Spheroid Culture and Sch B-Mediated Radiosensitization Assay

Single-cell suspensions of BC cells were prepared and mixed with Matrigel (Corning, NY, USA, 356237) at a 1:1 (*v*/*v*) ratio on ice. Fifty-microliter droplets of Matrigel-cell mixture were gently dispensed into a 24-well plate preheated to 37 °C, followed by incubation in incubator for 30 min to allow complete gelation. After gel formation, 500 μL of pre-warmed 3D spheroid culture medium was added along the walls to avoid disrupting the Matrigel droplets (detailed medium composition provided in Table S1). For radiosensitization assays, 3D spheroids in the RT monotherapy group (8 Gy + 8 Gy) and Sch B-combined RT group (40 μM Sch B + 8 Gy + 8 Gy) were irradiated with X-rays 8 Gy daily, for a total of 2 d, using 6 MV (megavolt) energy and a dose rate of 600 cGy/min. Subsequently, spheroids in Sch B monotherapy group and Sch B-combined RT group were added with 40 μM Sch B, with fresh medium supplemented with Sch B replaced on day 3. Six days post-irradiation, 3D spheroid viability was assessed by using the CellTiter-Glo 3D viability assay (Promega, Madison, WI, USA, G9683). Luminescence intensity was assessed by microplate reader (Bio-Rad).

### 4.5. Flow Cytometry

Cells were divided into control group (0 μM Sch B) and Sch B treatment groups (with final concentrations 10~60 μM) and incubated for 48 h under standard culture conditions. The cells were harvested and washed twice with pre-cooled PBS to remove residual medium. 4 mL of pre-cooled 70% ethanol was added overnight at −20 °C. The cell pellets were washed once with PBS to eliminate ethanol residue, then resuspended in ice-cold PBS and adjusted to a concentration of 1 × 10^6^ cells per sample. A total of 500 µL of PI/RNase staining solution was added to each sample, incubating in the dark for 15 min (with gentle mixing every 5 min to ensure uniform staining) and analyzed using BD flow cytometer.

### 4.6. Immunofluorescence Assay for γ-H2AX Foci Detection

Cells were seeded onto sterile coverslips in 24-well plates (5 × 10^4^ cells/well) allowed to adhere overnight, then pretreated with 40 μM Sch B for 4 h, and exposure to 4 Gy RT (6 MV energy, 600 cGy/min). At 4 h and 12 h post-RT, they were fixed with 4% paraformaldehyde for 15 min at 4 °C. Nonspecific binding was blocked by incubating cells with 5% BSA for 1 h, then incubated with primary anti-γ-histone-H2AX antibody (CST, Danvers, MA, USA, 2577s) diluted 1:300 overnight at 4 °C in a humidified chamber to prevent antibody evaporation, followed by incubated with Alexa488-conjugated secondary antibody (Abbkine, Wuhan, China, a23210) diluted 1:200 and incubated for 1 h. Nuclei were counterstained with DAPI for 5 min. Slides were mounted with antifade mountant. Z-stack fluorescence images were acquired using a fluorescent confocal microscope (Olympus Optical Co., Tokyo, Honshu, Japan) with sequential imaging along the *z*-axis. For each experimental group, at least 20 independent fields of view were captured with clearly resolvable γ-H2AX foci were selected for quantitative analysis. γ-H2AX foci were quantified using Fiji-ImageJ software 1.54f. The total number of foci was counted and normalized to the total number of DAPI-stained nuclei, and the average number of foci per nucleus was calculated from a minimum of 100 cells per group to ensure statistical reliability.

### 4.7. Real-Time PCR

Total RNA was extracted from harvested blood using the Blood Total RNA Extraction Kit (Servicebio, Wuhan, China, G3636-50T). Complementary DNA (cDNA) was synthesized with the ReverTra Ace qPCR RT Kit (Toyobo, Osaka, Japan, FSQ-101). qRT-PCR was performed using the SupRealQ Purple Universal SYBR qPCR Master Mix (Vazyme, Nanjing, China, Q412) on the X960 Real-Time PCR system (Heal Force, Shanghai, China). Specific primers for each target gene were designed via the Primer-BLAST tool on the NCBI website. All qPCR reactions were conducted in a 20 µL volume as the manufacturer’s guidelines. The primer sequences and amplification conditions are provided in Table S2. Gene expression levels were normalized to mouse β-actin using the 2^−ΔΔCT^ method.

### 4.8. Immunohistochemical Analysis

Tissues were subjected to routine fixation, paraffin embedding and sectioning, with the procedures performed as described previously [51]. Sections were incubated with anti-CD3 (1:400; Santa Cruz, CA, USA, sc-20047), anti-CD8α (1:400; CST, 98941s) overnight, and incubated with secondary antibodies (MXB Biotechnologies, KIT-9720) for 10 min, then developed with a DAB chromogenic solution (MXB Biotechnologies, Fuzhou, China, DAB-0031). The nuclei were counterstained with hematoxylin. Images were captured using an TEKSQRAY Slide Scanning System SQS-40P (TEKSQRAY, Beijing, China).

### 4.9. Animal Studies

#### 4.9.1. Animal Grouping and Drug Administration

Thirty-six female BALB/c mice (4~6 weeks, weighing 20 ± 2 g) were bought from Liaoning Changsheng Biotechnology Co., Ltd (Benxi, China). Experiments were performed following approval by the Animal Welfare and Ethics Committee of Affiliated Zhongshan Hospital of Dalian University (Approval No. DW2025-011-01). 4T1 cells (2 × 10^5^ cells per mouse) were subcutaneously inoculated into the mouse left hind limb. Tumor volume was monitored every two days, and the model was deemed successful when the volume reached ≥ 50 mm^3^. Tumor volume = length (L) × width^2^ (W^2^) × 0.5. The mice were randomly divided into six groups (*n* = 5 per group): (1) Untreated control group, (2) Model group, (3) Paclitaxel group (Abmole, Houston, TX, USA, M1970) (100 mg/kg), (4) Low-dose Sch B group (20 mg/kg), (5) Medium-dose Sch B group (40 mg/kg), (6) High-dose Sch B group (80 mg/kg). PTX and Sch B were dissolved in a mixture of 50% PEG300 and 50% normal saline. PTX was administered via oral gavage every 4 d and Sch B was every 2 d. The untreated control and model control groups received 200 µL saline *via* oral gavage every 2 d. Drug administration was continued for 14 consecutive days. Body weight were monitored regularly throughout the experiment.

#### 4.9.2. Collection and Preparation of Samples

Following anesthesia, blood was collected via eyeball enucleation and the mice were euthanized. Tumors were excised and photographed immediately. One half of each tumor was fixed in 4% paraformaldehyde, while the other half was stored at −80 °C for molecular biological assays. Intestinal contents were also collected and stored at −80 °C.

### 4.10. 16S rDNA Sequencing

Total genomic DNA was isolated from mouse cecal content samples. DNA concentration and purity were quantified with A260/A280 ratios of 1.8–2.0. The V3-V4 hypervariable regions of the bacterial 16S rRNA gene were amplified by PCR using barcoded forward primer 341F (5′-CCTAYGGGRBGCASCAG-3′) and reverse primer 806R (5′-GGACTACHVGGGTWTCTAAT-3′). The barcodes ensured sample differentiation during high-throughput sequencing. PCR amplification conditions are provided in Table S2. PCR amplicons were verified to confirm target band sizes, purified to remove non-specific products and impurities, and normalized to a uniform concentration. High-throughput sequencing was performed on the Illumina MiSeq PE300 platform. Raw sequencing reads were subjected to strict quality filtering to exclude low-quality reads and adapter-contaminated reads. Operational Taxonomic Units (OTUs) were clustered at 97% similarity, and taxonomic annotation was conducted against the Silva database for species classification. Based on the OTU data, microbiota composition profiling at the phylum and genus levels, as well as quantification of gut microbiota species abundance, were carried out. The associations among microbial genera, SCFA levels, and immune-related biochemical indices were evaluated by spearman’s rank correlation analysis.

### 4.11. Detection of SCFA Content

Approximately 100 mg of mouse cecal contents was weighed and transferred into a grinding tube containing methanol, followed by thorough homogenization. The homogenized mixture was centrifuged at 14,000× *g* for 20 min at 4 °C to precipitate insoluble debris. An aliquot of 10 μL supernatant was mixed with 10 μL precipitant supplemented with internal standard, and the mixture was vortexed for 5 min. The supernatant was incubated at 40 °C for 30 min to undergo derivatization with 3-Nitrophenylhydrazine (3-NPH) and 1-Ethyl-3-(3-dimethylaminopropyl)carbodiimide (EDC). The derivatization reaction was terminated by adding 0.5% ultrapure water. A 5 μL aliquot of the final supernatant was subjected to LC-MS/MS analysis on a Shimadzu system, equipped with a C18 chromatographic column maintained at 40 °C. The mobile phase consisted of 0.1% formic acid in water (phase A) and acetonitrile (phase B), with a gradient elution program: 10% B from 0 to 14 min, linear gradient from 45% to 80% B between 14 and 16 min, constant 80% B from 16 to 18 min, followed by re-equilibration at 10% B for 2 min. Mass spectrometry was operated in ESI^−^ mode, with a capillary voltage of −4.5 kV and desolvation temperature of 450 °C, to quantify acetate, propionate, butyrate, isobutyrate, valerate, and isovalerate via their 3-NPH derivative MRM transitions. Calibration curves were constructed by spiking SCFAs standard solutions into blank cecum content matrix, and the concentrations of SCFAs in samples were calculated against these calibrated curves. Differential metabolites were screened based on the criteria of *p* ≤ 0.05 and fold change ≥ 1.2.

### 4.12. Statistical Analysis

Experimental data were analyzed using GraphPad Prism software 8.4.3. Data are presented as the mean ±SD. Student’s *t*-test was employed for pairwise comparisons between two groups to evaluate statistical differences. A two-tailed *p* value < 0.05 was considered to indicate significance; *n* ≥ 3 for all cell and 3D spheroid experiments, *n* = 5 for mouse tumor-volume measurements and SCFA detection; *n* = 3 for 16S rDNA sequencing; and *n* = 6 for IHC quantification. Spearman’s correlation was used for associations among microbial genera, SCFAs, and immune markers. False discovery rate (FDR) correction was applied to control for multiple comparisons and reduce false-positive results.

## 5. Conclusions

Our study revealed that Sch B is a promising natural radiosensitizer with dual direct mechanisms and gut microbiota-mediated immunomodulatory effects. Its favorable safety profile and multidimensional action make it a potential candidate for clinical translation, offering a new therapeutic strategy for improving the efficacy of BC radiotherapy.

## Data Availability

All data generated or analyzed during this study are included in this published article. The original data supporting these findings are available at any time upon request to the corresponding author.

## Supplementary Materials

The following supporting information can be downloaded at: https://www.mdpi.com/article/10.3390/ph19060883/s1, Table S1: Composition of culture medium for breast cancer 3D spheroids; Table S2: Primer sequences and amplification conditions in PCR experiments. Figure S1: Experimental design and drug administration schedule for in vivo studies and radiosensitization assays in tumor 3D spheroid.

## Abbreviations

The following abbreviations are used in this manuscript:

3-NPH	3-Nitrophenylhydrazine
BC	Breast cancer
CCK-8	Cell counting kit-8
CDK4/6	Cyclin-dependent kinase 4/6
CXCL10	C-X-C motif chemokine ligand 10
DSBs	DNA double-strand breaks
EDC	1-Ethyl-3-(3-dimethylaminopropyl)carbodiimide
F/B Ratio	Firmicutes/bacteroidetes ratio
IFN-γ	Interferon-γ
IHC	Immunohistochemical analysis
IL-18	Interleukin-18
OUT	Operational taxonomic units
PE	Plating Efficiency
PTX	paclitaxel
RT	Radiotherapy
SCFAs	Short-chain fatty acids
Sch B	Schisandrin B
SER	Sensitization enhancement ratio
SF	Surviving fraction
TCM	Chinese medicine
