# Supplementary material for: Schisandrin B Exerts Radiosensitizing Effects on Breast Cancer via Dual Mechanisms of Cell Cycle/DNA Repair and Gut Microbiota-Immune Axis Modulation
Source: Pharmaceuticals (Basel). 2026 Jun 1;19(6):883. doi: 10.3390/ph19060883 (PMC13304617; doi:10.3390/ph19060883)
Supplement: Supplementary file 1 [file pharmaceuticals-19-00883-s001.zip › Fig S1.pdf]

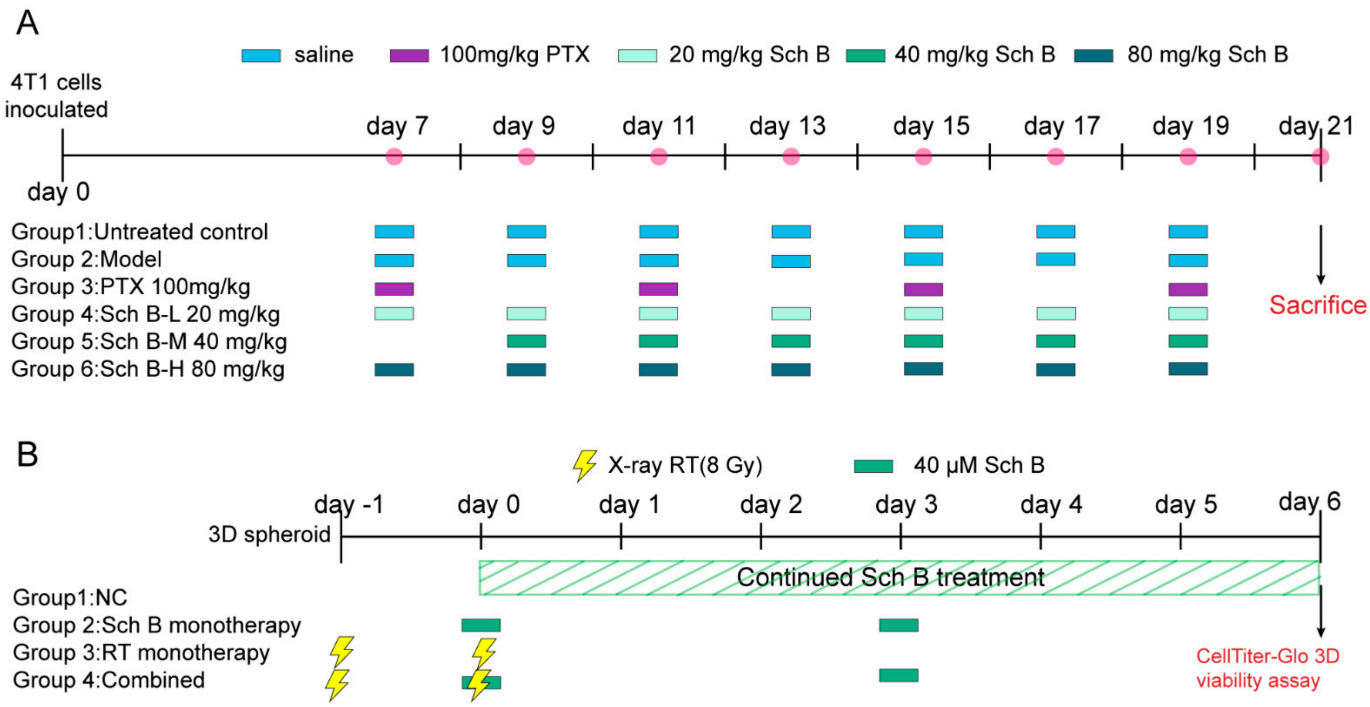

Figure S1. Experimental design and drug administration schedule for in vivo studies and radiosensitization assays in tumor 3D spheroid. (**A**) Mice were randomly divided into 6 groups (n=6 per group): untreated control, model, PTX, and low/medium/high-dose Sch B groups. PTX and Sch B were dissolved in 50% PEG300-normal saline. PTX was administered orally every 4 days, Sch B every 2 days; the control and model groups received normal saline. All groups were treated for 14 consecutive days (n=5). **(B)** RT monotherapy and combined group received 8 Gy X-ray daily for 2 days. Sch B monotherapy and Sch B-combined RT group was treated with 40 μM Sch B after irradiation. Medium with 40 μM Sch B was refreshed on day 3. Spheroid viability was detected by CellTiter-Glo 3D Assay 6 days post-irradiation (n=3).
