# Supplementary material for: Schisandrin B Exerts Radiosensitizing Effects on Breast Cancer via Dual Mechanisms of Cell Cycle/DNA Repair and Gut Microbiota-Immune Axis Modulation
Source: Pharmaceuticals (Basel). 2026 Jun 1;19(6):883. doi: 10.3390/ph19060883 (PMC13304617; doi:10.3390/ph19060883)
Supplement: Supplementary file 1 [file pharmaceuticals-19-00883-s001.zip › Table S1.pdf]

Table S1 Composition of culture medium for breast cancer 3D spheroid

Reagents	Supplier	Catalogue number	Final Concentration
R-Spondin-3	Peprotech	120-44	250 ng/mL
Noggin	Peprotech	120-10C	100 ng/mL
FGF10	Peprotech	100-26	20 ng/mL
FGF7	Peprotech	100-19	5 ng/mL
EGF	Peprotech	AF-100-15	5 ng/mL
Heregulin β1	Peprotech	100-03	5 nM
A83-01	Tocris	2939	500 nM
SB202190	Sellerk	S1077	500 nM
B27 supplement	Gibco	17504-44	1×
Nicotinamide	Sigma	N0636	10 mM
N-Acetylcysteine	Sigma	A9165	1.25 mM
Primocin	Ant-pm-1	Invivogen	50 µg/mL
AdDF++++	-	-	-
